# A Case-Control Study between Gene Polymorphisms of Polyunsaturated Fatty Acid Metabolic Rate-Limiting Enzymes and Acute Coronary Syndrome in Chinese Han Population

**DOI:** 10.1155/2013/928178

**Published:** 2013-02-28

**Authors:** Zikai Song, Hongyan Cao, Ling Qin, Yanfang Jiang

**Affiliations:** Department of Cardiology, the Second Division of the First Hospital, Jilin University, 3302 JiLin Street, Changchun 130031, China

## Abstract

The purpose of this study is to analyze the relationship between the polymorphisms of fatty acid desaturase 1 (*FADS*1), fatty acid desaturase 2 (*FADS*2), and elongation of very long-chain fatty acids-like 2 (*ELOVL*2) and acute coronary syndrome (ACS) in Chinese Han population. Therefore, we selected three single nucleotide polymorphisms (SNPs) from these candidate genes and genotyped them using PCR-based restriction fragment length polymorphism analysis in 249 ACS patients and 240 non-ACS subjects, as were Han Chinese ancestry. The results showed that rs174556 in the *FADS*1 gene is found to be in allelic association (*P* = 0.003 ) and genotypic association (*P* = 0.036) with ACS. The frequencies of rs174556 minor allele (T) in case group were obviously higher than in control group. The trans-phase gene-gene interaction analysis showed that the combined genotype of rs174556 (T/T) and rs3756963 (T/T) was associated with ACS (*P* = 0.031). And the results suggest that, for rs174556 C>T, the CT/TT genotypes were more likely to lead in ACS in subjects with hypertension after correction of all risk factors (OR = 4.236, 95% CI, 2.216–7.126). These findings suggest that the polymorphisms of rs174556 in the *FADS*1 gene are very likely to be associated with ACS in Chinese Han population, especially in subjects with hypertension.

## 1. Introduction

Acute coronary syndrome (ACS) is a common disease, and a major determinant of morbidity and mortality in all races, ethnicities, and cultures [1], which is caused by a combination of genetic background and environmental factors. The epidemic of coronary artery disease (CAD), especially its manifestation as ACS, is a global issue that accounts for more than 80% of the burden of this disease in developing countries [2] and results in approximately 30% of all deaths worldwide each year [3]. The spectrum of ACS ranges from unstable angina pectoris (UAP) to acute myocardial infarction (AMI), including ST-segment elevation myocardial infarction (STEMI) and non-STEMI (NSTEMI) [4]. Atherosclerotic plaque instability is the main feature of ACS pathogenesis.

Blood and tissue contents of polyunsaturated fatty acid (PUFA) and long-chain PUFA (LC-PUFA) are related to numerous health outcomes including cardiovascular health, allergies, mental health, and cognitive development [5]. There are two families of PUFA, and they are classified as omega-3 (n-3) and omega-6 (n-6) based on the location of the last double bond relative to the terminal methyl end of the molecule [6]. Evidence from various research paradigms supports the cardiovascular benefits of a high intake of n-3 polyunsaturated fatty acids (PUFAs), especially the long-chain, marine-derived n-3 PUFA, eicosapentaenoic acids, and docosahexaenoic acids [7, 8]. And n-6 PUFA are well known for their critical role in many physiological functions and seem to reduce risks of CAD [9].

Both families of fatty acids, n-3 and n-6, share and compete for the same enzymes (Δ6-desaturase, Δ5-desaturase, and elongases) in their biosynthesis, and Δ6-desaturase is the rate-limiting step [10–12]. Δ5-desaturase (D5D) and Δ6-desaturase (D6D) are encoded by the genes *FADS1* and *FADS2*, respectively, which form a gene cluster jointly with the gene for fatty acid desaturase 3 (*FADS3*) on the human chromosome 11q12-q13.1 [12, 13]. And D5D and D6D mainly involved in regulating the levels of proinflammatory and anti-inflammatory eicosanoids derived from PUFAs [14]. Additionally, another essential enzyme, elongase, is involved in the homeostasis of longer chain n-3 fatty acids, which is encoded by elongation of very long-chain fatty acids-like 2 (FEN1/Elo2, SUR4/Elo3, yeast) (*ELOVL2*) gene [15].

Polymorphisms in the genes *FADS1* and *FADS2* are associated with n-3 and n-6 fatty acid levels and especially with arachidonic acid (ARA) amounts in blood and several tissues [16–22]. The presence of a variant T to deletion (T-del) in the promoter of the Δ6-desaturase gene (*FADS2*) leads to reduced timnodonic acid (EPA) concentrations in plasma and adipose tissue [18], suggesting that this variant decreases enzyme activity and therefore conversion from ALA. The presence of the *FADS2* T-del variant is also associated with higher plasma triglyceride concentrations [18]. Additionally, a number of studies have reported significant associations between *FADS* genotypes and the risk of CAD [23, 24]. In CHIANTI study, the genome-wide association study (GWAS) showed that the strongest evidence for association was observed in a region of chromosome 11 that encodes three fatty acid desaturases (*FADS1*, *FADS2*, and *FADS3*) [25]. In 2010, another large GWAS repeated the strongest association between *FADS1* and *ELOVL2* genes and the ratio of product to precursor fatty acids [26]. Several studies, including a recent meta-analysis of genome-wide association (GWA) scans, confirmed that polymorphisms in the *FADS* gene cluster were associated with PUFA concentrations in serum phospholipids and erythrocyte cell membranes in several populations, including Caucasians, East Asians, and African Americans [19, 23, 25, 27–29].

Until now, it is unknown whether or not SNPs in the *FADS1*/*FADS2* gene cluster and *ELOVL2* gene are associated with ACS in Chinese Han population. The aim of this study was to investigate the possible association between the gene SNPs of PUFA metabolic rate-limiting enzymes and risk of ACS in Chinese Han population through the case-control study containing *FADS1*/*FADS2* and *ELOVL2* genes.

## 2. Subjects and Methods

### 2.1. Study Subjects

This case-control study included 249 ACS patients and 240 controls in order to undertake a genetic analysis for association between the PUFA rate-limiting enzymes gene polymorphisms and ACS. All patients used for this study were Chinese of Han descent. Patients with ACS were admitted to the First Hospital of Jilin University, Changchun, China, in the period between 2008 and 2010. All subjects gave written informed consent for the study. The study was approved by the ethics committee of Jilin University, Changchun, China.

Diagnosis was carried out independently by at least two well-trained physicians based on the following criteria. All patients (143 males and 106 females) were identified with ACS by coronary computed tomographic angiography (SIEMNS Somatom Definition AS + 128 row spiral CT). ACS was defined by ≥75% stenosis in any major coronary artery. Acute coronary syndrome encompasses three clinical diagnoses: unstable angina, non-ST-segment elevation myocardial infarction, and ST-segment elevation myocardial infarction. Myocardial infarction with cardiac chest pain, serologic evidence of myonecrosis, and persistent (>20 min) ST-segment elevation was confirmed as WHO criteria issued in 1979 [30]; the definition of UAP is when cardiac chest pain was new or worsening without serologic evidence of myonecrosis (i.e., no elevation of serum troponin or creatine kinase MB isoenzyme concentration), or dynamic electrocardiographic (ECG) changes (i.e., ST depression and/or T wave inversion); the definition of NSTEMI is when the patient had cardiac chest pain and serologic evidence of myocardial necrosis in the absence of ST-segment elevation on the ECG [31], and patients with nonatherosclerotic vascular diseases, congenital heart disease, cardiomyopathy, valvular disease, renal or hepatic disease, and cancer were excluded. Based on the principle of epidemiology for setting control group [32], control group contained 240 healthy patients (126 males and 114 females) randomly selected from the same geographical area who were undergoing a routine checkup as part of annual physical examination, which included an ECG, chest X-ray, and serum analysis. The control group was formed on the basis of their unremarkable physical examination, as well as the absence of personal or family history and reasons to suspect ACS.

The presence of cardiovascular risk factors, including diabetes mellitus, blood pressure, and cigarette smoking, was obtained from all participants. Diagnosis of hypertension and diabetes mellitus was performed according to World Health Organization criteria. In this study, hypercholesterolemia was defined as a serum total cholesterol level of 200 mg/dL or more, and a smoking habit was defined as a daily intake of >10 cigarettes [4]. Overnight fasting venous blood samples were collected from all subjects for genomic DNA extraction.

### 2.2. Genotyping

Three SNPs rs174556 (Mbo I site) in the *FADS1*, rs174617 (Msp I site) in the *FADS2*, and rs3756963 (Hha I site) in the *ELOVL2* were selected as genetic markers. They were all C to T base change present in intron. SNP information was obtained from NCBI dbSNP Build 132 (http://www.Ncbi.nlm.nih.gov/SNP/). The candidates SNPs were restricted to minor allele frequency bigger than 15% in HAPMAP-CHB database (http://www.hapmap.org/). Genomic DNA used for PCR amplification was extracted from the whole blood sample using a DNA extraction kit (Promega, Beijing, China). SNPs were genotyped using standard polymerase chain reaction and restriction fragment length polymorphism (RFLP) analysis. The sequences of primers for amplification are available as follows (Table 1). PCR conditions included predenaturation at 94°C for 5 minutes followed by 35 cycles of 95°C for 35–40 seconds, 63–57°C for 1 minutes, and 72°C for 1 minutes, and a final extension at 72°C for 10 minutes.

### 2.3. Statistical Analysis

Data were expressed as percentages of total categorical variables, or mean ± SD. The statistical analyses on the characteristics of the subjects were performed with Pearson *χ* ^2^ test for the categorical variables such as sex, smokers, and nonnormal distribution variable age was compared by Mann-Whitney rank sum test.

The Hardy-Weinberg equilibrium for the genotypic distributions of SNPs was tested using the Chi-square (*χ* ^2^) goodness-of-fit test. The Haploview program (version 4.1) was applied to estimate the linkage disequilibrium (LD) measures (*D*′ and *r* ^2^) between paired SNPs. Allelic association, genotypic association, and analysis for gene-gene interaction were performed with the UNPHASED program, which is an application for performing genetic association analysis in nuclear families and unrelated subjects [21]. Results are expressed as odds ratio (OR) and 95% confidence intervals (CI). *P* values < 0.05 were considered significant. With regard to the gene-gene interaction tests, the genotypes with relative frequencies of less than 3% were not considered for analysis. We also applied the permutation test (1000 times) performed with UNPHASED to correct the final *P* values for the markers used.

## 3. Results

### 3.1. Characteristics of Study Subjects

Table 2 lists the demographic and clinical characteristics of the 249 ACS patients and 240 control subjects. Compared with control group, ACS group had more males, more smokers, and more individuals with diabetes. However, there was no significant difference of the mean age and proportion of hypertension between case and control groups.

### 3.2. Allele and Genotype Analysis

Rs174556 of *FADS1*, rs174617 of *FADS2*, and rs3756963 of *ELOVL2* were genotyped, and they all lie within intron. Rs174556 and rs174617 locate in different LD block on 11q12-q13.1 region (*D*′ = 0.57, *r* ^2^ = 0.52). Rs3756963 locates on 6p24.2. The *χ* ^2^ goodness-of-fit test showed that the genotypic distributions of rs174556, rs174617, and rs3756963 were not deviated from Hardy-Weinberg equilibrium in both case and control groups (*P* > 0.05). Tables 3 and 4 present the distributions of alleles and genotypes of 3 SNPs among participants, respectively. For rs174556, C was major allele, frequency was 60.6% and 69.6% in case and control group, respectively, but for rs174617 and rs3756963, T was major allele.

Analysis with the UNPHASED software showed that rs174556 was associated with ACS before (*χ* ^2^ = 8.592, *P* = 0.003) and after 1000 permutation test (*P* = 0.003996), and frequency of minor allele T of rs174556 was significantly higher in case than control subjects (Table 3). However, rs174617 and rs3756963 were not associated with ACS. As shown in Table 4, the logistic regression analysis test revealed genotypic association between rs174556 and ACS after being adjusted for confounding factors (*χ* ^2^ = 6.084, *P* = 0.036), but not the genotypic association was observed between the other 2 SNPs and ACS.

We further analyzed the associations between the polymorphisms of three SNPs and ACS for subgroups with or without hypertension, DM, and smoking. The results suggest that, for rs174556 C>T, compared with the CC genotype, the CT/TT genotypes were more likely to result in ACS in subjects with hypertension after correction of all risk factors (OR = 4.236, 95% CI, 2.216–7.126) (Table 5). Whereas, another two SNPs, rs174556 and rs3756963, were not associated with ACS after correction of all risk factors (Table 6).

### 3.3. Trans-Phase Gene-Gene Interaction Analysis

In Table 7, the combined genotypes for rs174556 and rs3756963 with frequency more than 3% are presented. We used the most commonly combined genotype major homozygote as a reference, and the results showed that *ELOVL2* gene had a combined effect with *FADS1* gene (*χ* ^2^ = 14.112, df = 6, *P* = 0.028). Rs174556 (C/C)-rs3756963 (T/T) and rs174556 (T/T)-rs3756963 (T/T) were associated with ACS (*χ* ^2^ = 4.478, *P* = 0.034, *χ* ^2^ = 4.656, *P* = 0.031).

### 3.4. Logistic Regression Analysis

As shown in Table 8, according to a multivariate logistic regression analysis, the most predictive risk factor for ACS was hypertension, followed by smoking, diabetes, and the T allele in rs174556. The T allele in rs174556 may be a risk factor for ACS (OR = 1.791, 95% CI, 1.088–2.951).

## 4. Discussion

*FADS1*, *FADS2*, and *ELOVL2* all encode rate-limiting enzymes in PUFA metabolism. And many studies have confirmed that high levels of PUFA in plasma phospholipids, cell membranes, or whole blood were associated with lower risk of multiple diseases, including metabolic syndrome, CAD, et al. Therefore, we investigate the association between the common variants of the three genes and ACS.

Our results show that the frequency of minor allele T of rs174556 was remarkably higher in case than control group (*P* = 0.003), as the frequency of TT genotype of rs174556 in case group was also markedly higher than in control group (*P* = 0.036). And for rs174556 C>T, compared with the CC genotype, the CT/TT genotypes were more likely to lead to ACS in subjects with hypertension after correction of all factors. Rs174617 in the *FADS2* was not associated with ACS. Based on our results, therefore, rs174556 in the *FADS1* has a significant role in the development of ACS, especially in subjects with hypertension. But the locus rs174556 is not a functional SNP, which located in intron of *FADS1* gene. As a result, we infer that the *FADS1* gene may confer susceptibility of ACS through affecting nearby gene. Many studies also have proved that SNPs in *FADS1*/*FADS2* were associated with plasma lipid concentrations in adult populations and children [33, 34]. However, in contrast with our results, Aslibekyan et al. [35] failed to show relationship between rs174556 and MI in the Costa Rica Study. Firstly, this discrepancy may be due to racial differences in two studies. Secondly, the research objects are different. Finally, the difference of sample size also may be another possible reason.

*FADS2* is located in chromosome 11 and expressed in almost all human tissues, especially in the liver, heart, and brain [36]. Some studies have report the metabolic effects of polymorphisms in this gene or their effects on the risk of CAD [24]. But the locus rs174617 of *FADS2* is rarely reported. In 2011, our previous study showed that this locus was not associated with CAD [37], which is consistent with our present study. The reason may be that a large proportion of regulatory SNPs, which affect gene expression, are located in the promoter regions [38].

At present, rs174537 near *FADS1* is considered to be the most relevant locus with ARA. GWAS in the In CHIANTI Study showed that minor allele homozygotes rs174537 (TT) had lower plasma concentrations of ARA compared to major allele homozygotes [25]. And a study in Korea population confirmed that rs174537 T had lower proportions of ARA in serum phospholipids and reduced CAD risk [24]. Many studies suggested the minor allele carriers including rs174556 T may have lower desaturase activity [25, 37]. Therefore, based on above results, a possible causality link between lower desaturase activity and vascular disease has been suggested [39]. However, at present, the further research of gene function is still lacking.

*ELOVL2* is a member of the mammalian microsomal *ELOVL* fatty acid enzyme family, involved in the elongation of very long-chain fatty acids including PUFAs required for various cellular functions in mammals [40]. A GWAS study found that an association of EPA with variants in the *FADS1* gene reached genome-wide significance level, and independent follow-up investigation showed associations of a selected *FADS1* variant with erythrocyte membrane levels of EPA, ALA, and DPA and of an *ELOVL2* variant with DPA and DHA [25]. These findings confirm an influence of *FADS1* and *ELOVL2* on selected n-3 PUFAs. In contrast, we do not found the association between rs3756593 of *ELOVL2* gene and ACS. Both allele and genotype analysis all have no statistical significance, but the trans-phase gene-gene interaction test revealed that the* ELOVL2* gene combined with *FADS1* gene had an effect on ACS. Above result implies that the *FADS1* gene-*ELOVL2* gene interaction may affect PUFA concentrations and afford susceptibility to ACS. But the exact mechanisms for the interaction are currently unknown.

In conclusion, this case-control study preliminary indicates that variations in *FADS1* may affect the risk of ACS and provide a genetic basis of molecular biology. We will continue to analyze the gene functional and the serum levels of phospholipids fatty acid in ACS.

## Figures and Tables

**Table 1:** The sequences of primers for amplification.

Genes	SNPs	Primers	Sequences
*FADS1 *	rs174556	Forward	5′AAGCAGGGACCTCAAGAC3′
		Reverse	5′AGCCCACCAAGAATGTAA3′
*FADS2 *	rs174617	Forward	5′GAACTGTCAGAGGCAACG3′
		Reverse	5′CTGGGCAATAAAGCAAGA3′
*ELOVL2 *	rs3756963	Forward	5′CCCTTTGTGCGAGAACCT3′
		Reverse	5′ATCCCAAGCGACAGACCC3′

**Table 2:** General characteristics of patients and controls included in our study.

Subject characteristics	Case	Control	*P* value
	(*n* = 249)	(*n* = 240)	
Age (years)	62.00 ± 17.00	63.00 ± 23.70^a^	0.148
Male, *n* (%)	143 (57.4)	126 (52.5)	0.273
Diabetes, *n* (%)	86 (34.5)	30 (14.4)	<0.001
Hypertension, *n* (%)	154 (61.8)	96 (40.0)	<0.001
Smokers, *n* (%)	162 (65.1)	86 (35.8)	<0.001

^a^Median ± QR.

Age was compared by using Mann-Whitney rank sum test.

Male, diabetes, hypertension, and smokers were compared by using Pearson's Chi-square test.

**Table 3:** Distribution of allele frequencies of SNPs in case and control groups.

Genes	SNPs	Allele	Case (%)	Control (%)	*χ* ^2^	*P*	OR	95% CI
*FADS1 *	rs174556	C	302 (60.6)	334 (69.6)	8.592	0.003^a^	1.485	1.139–1.935
		T	196 (39.4)	146 (30.4)				
*FADS2 *	rs174617	T	386 (77.5)	380 (79.2)	0.395	0.530	1.103	0.813–1.495
		C	112 (22.5)	100 (20.8)				
*ELOVL2 *	rs3756963	T	368 (73.9)	372 (77.5)	1.725	0.189	1.217	0.908–1.631
		C	130 (26.1)	108 (22.5)				

^a^The adjusted *P* value was 0.003996 from 1000 permutations.

**Table 4:** Distribution of genotype frequencies of SNPs in case and control groups.

Genes	SNPs	Genotype	Case (%)	Control (%)	*χ* ^2^	*P*	OR	95% CI
*FADS1 *	rs174556	C/C	96 (38.6)	119 (49.6)	6.084	0.036^a^		
		C/T	110 (44.2)	96 (40.0)			0.826^a^	0.418–1.459^a^
		T/T	43 (17.3)	25 (10.4)			0.324^a^	0.126–0.864^a^

*FADS2 *	rs174617	T/T	148 (59.4)	149 (62.1)	0.410	0.815		
		C/T	90 (36.1)	82 (34.2)			1.105	0.759–1.609
		C/C	11 (4.4)	9 (3.8)			1.230	0.495–3.056

*ELOVL2 *	rs3756963	T/T	141 (56.6)	145 (60.4)	2.301	0.317		
		C/T	86 (34.5)	82 (34.2)			1.079	0.737–1.579
		C/C	22 (8.8)	13 (5.4)			1.740	0.844–3.589

^a^Adjustment for age, sex, and the presence of diabetes, hypertension, and smoking by forward logistic regression analysis.

**Table 5:** Stratified analysis between the rs174556 C>T polymorphisms and risk of ACS by hypertension, DM, and smoking.

	Case (*n* = 249)		Control (*n* = 240)		Adjusted OR (95% CI)^a^	
	CC (%)	CT + TT (%)	CC (%)	CT + TT (%)	CC	CT + TT
Rs174556 C>T genotypes						

Hypertension						
No	24 (25.3)	71 (74.7)	43 (29.9)	101 (70.1)	1.00	2.640 (1.410–4.560)
Yes	72 (46.8)	82 (53.2)	76 (79.2)	20 (20.8)	1.00	4.236 (2.216–7.126)
DM						
No	26 (16.0)	137 (84.0)	101 (48.1)	109 (51.9)	1.00	4.120 (2.326–7.824)
Yes	70 (81.4)	16 (18.6)	18 (60.0)	12 (40.0)	1.00	0.424 (0.182–0.926)
Smoking						
No	10 (11.5)	77 (88.5)	49 (31.8)	105 (68.2)	1.00	3.624 (1.842–7.636)
Yes	86 (53.1)	76 (46.9)	70 (81.4)	16 (18.6)	1.00	0.820 (0.326–1.726)
No 3 risk	10 (16.1)	52 (83.9)	36 (32.1)	76 (67.9)	1.00	2.016 (1.046–5.236)

^a^Ajusted for age, sex, and the presence of diabetes, hypertension, and smoking status except the stratified factor at each stratum.

**Table 6:** Stratified analysis between the rs174617 and rs3756963 T>C polymorphisms and risk of ACS by hypertension, DM, and smoking.

	Case (*n* = 249)		Control (*n* = 240)		Adjusted OR (95% CI)^a^	
	TT (%)	CC + CT (%)	TT (%)	CC + CT (%)	TT	CC + CT
Rs174617 T>C genotypes						

Hypertension						
No	51 (58.0)	37 (42.0)	109 (75.7)	35 (24.3)	1.00	2.326 (1.327–4.120)
Yes	90 (58.4)	64 (41.6)	40 (41.7)	56 (58.3)	1.00	0.492 (0.262–0.812)
DM						
No	88 (54.0)	75 (46.0)	135 (64.3)	75 (35.7)	1.00	1.242 (1.006–2.012)
Yes	60 (69.8)	26 (30.2)	14 (46.7)	16 (53.3)	1.00	0.326 (0.126–0.801)
Smoking						
No	58 (82.8)	29 (17.2)	113 (73.4)	41 (26.6)	1.00	0.524 (0.326–0.884)
Yes	90 (46.9)	72 (53.1)	36 (41.9)	50 (58.1)	1.00	0.628 (0.412–1.021)
No 3 risk	50 (71.4)	20 (28.6)	60 (65.2)	32 (34.8)	1.00	0.782 (0.382–1.410)

Rs3756963 T>C genotypes						

Hypertension						
No	67 (70.5)	28 (29.5)	115 (79.9)	29 (20.1)	1.00	1.542 (0.743–2.732)
Yes	74 (48.1)	80 (51.9)	30 (31.2)	66 (68.8)	1.00	0.524 (0.301–0.980)
DM						
No	89 (16.0)	74 (84.0)	125 (59.5)	85 (40.5)	1.00	1.364 (0.896–1.868)
Yes	52 (60.5)	34 (39.5)	20 (66.7)	10 (33.3)	1.00	0.692 (0.284–1.790)
Smoking						
No	59 (11.5)	28 (88.5)	105 (31.8)	49 (68.2)	1.00	0.423 (0.298–0.792)
Yes	82 (53.1)	80 (46.9)	40 (81.4)	46 (18.6)	1.00	0.920 (0.583–1.492)
No 3 risk	60 (85.7)	10 (14.3)	80 (74.1)	28 (25.9)	1.00	0.524 (0.301–1.162)

^a^Ajusted for age, sex, and the presence of diabetes, hypertension, and smoking status except the stratified factor at each stratum.

**Table 7:** The trans-phase analysis for genotypic combined effect in case and control groups.

Combined genotype^a^	Case	Control	*χ* ^2^	*P*	OR (95% CI)
rs174556 (C/C)-rs3756963 (T/T)	52	70	4.478	0.034	referent
rs174556 (C/C)-rs3756963 (C/T)	38	46	1.310	0.252	1.112 (0.635–1.946)
rs174556 (C/T)-rs3756963 (C/T)	30	34	0.482	0.487	1.188 (0.806–2.891)
rs174556 (C/T)-rs3756963 (T/T)	70	66	0.023	0.880	1.428 (0.873–2.335)
rs174556 (T/T)-rs3756963 (T/T)	21	9	4.656	0.031	3.141 (1.330–7.418)

Test of overall association: *χ* ^2^ = 14.112, df = 6, *P* = 0.028.

^a^Only listed the combined genotypes with frequencies >3%.

**Table 8:** The logistic regression analysis for the relation between the risk factors and ACS.

Risk	*P*	OR value	95% CI
Hypertension	0.000	4.114	2.224–7.611
Smoking	0.000	2.444	1.563–3.823
Diabetes	0.035	2.243	1.052–4.798
rs174556 T allele	0.022	1.791	1.088–2.951

Smoking versus nonsmoking, hypertension versus nonhypertension, DM versus non-DM, rs174556 T allele versus rs174556 C allele.
